# The HDAC inhibitor valproate induces a bivalent status of the CD20 promoter in CLL patients suggesting distinct epigenetic regulation of CD20 expression in CLL *in vivo*

**DOI:** 10.18632/oncotarget.16964

**Published:** 2017-04-08

**Authors:** Annarita Scialdone, Muhammad Sharif Hasni, Jesper Kofoed Damm, Andreas Lennartsson, Urban Gullberg, Kristina Drott

**Affiliations:** ^1^ Department of Hematology and Transfusion Medicine, Lund University, Lund, Sweden; ^2^ Department of Biosciences and Nutrition, Karolinska Institutet, Huddinge, Sweden; ^3^ Clinic of Oncology, Skåne University Hospital, Lund, Sweden

**Keywords:** valproate, chronic lymphocytic leukemia, H3K27me3, CD20, EZH2

## Abstract

Treatment with anti-CD20 antibodies is only moderately efficient in chronic lymphocytic leukemia (CLL), a feature which has been explained by the inherently low CD20 expression in CLL. It has been shown that CD20 is epigenetically regulated and that histone deacetylase inhibitors (HDACis) can increase CD20 expression *in vitro* in CLL. To assess whether HDACis can upregulate CD20 also *in vivo* in CLL, the HDACi valproate was given to three del13q/NOTCH1wt CLL patients and CD20 levels were analysed (the PREVAIL study). Valproate treatment resulted in expected global activating histone modifications suggesting HDAC inhibitory effects. However, although valproate induced expression of CD20 mRNA and protein in the del13q/NOTCH1wt I83-E95 CLL cell line, no such effects were observed in the patients studied. In contrast to the cell line, in patients valproate treatment resulted in transient recruitment of the transcriptional repressor EZH2 to the CD20 promoter, correlating to an increase of the repressive histone mark H3K27me3. This suggests that valproate-mediated induction of CD20 may be hampered by EZH2 mediated H3K27me3 *in vivo* in CLL. Moreover, valproate treatment resulted in induction of EZH2 and global H3K27me3 in patient cells, suggesting transcriptionally repressive effects of valproate in CLL. Our results suggest new *in vivo* mechanisms of HDACis which may have implications on the design of future clinical trials in B-cell malignancies.

## INTRODUCTION

Monoclonal antibodies targeting CD20 is one of the cornerstones in the treatment of B-cell malignancies. The primary mechanisms of action of CD20 antibodies include antibody-dependent cellular cytotoxicity (ADCC), complement-dependent cytotoxicity (CDC), and induction of apoptosis [1].

During recent years, the first generation monoclonal CD20 antibody rituximab has been followed by the second generation CD20 antibodies obinutuzumab and ofatumumab, with improved *in vitro* ADCC and CDC, respectively. Nevertheless, acquired or inherent resistance to anti-CD20 treatment is a remaining clinical obstacle. Downregulation of CD20 has been described in a number of case reports of patients with relapsed/refractory B-cell lymphoma who became unresponsive to rituximab-based therapies and is probably one of the most important factors contributing to rituximab-resistance [2, 3]. For example, Tsai et al reported reduced CD20 promoter activity and a defect in CD20 transport as two novel mechanisms responsible for CD20 downregulation in rituximab-resistant cell lines [4]. Moreover, Sugimoto and colleagues have shown escape from CD20 antibody treatment by CD20 downregulation mediated by recruitment of the Sin3A-HDAC1 complex to the CD20 promoter in resistant B-cell lymphoma cell lines [5]. This suggests that inhibitors of HDACs (HDACis) could counteract rituximab-resistance, and is consistent with the finding by our group that the HDACi valproate upregulates CD20 protein and mRNA expression *in vivo* in diffuse large B-cell lymphoma (DLBCL) patients [6]. Moreover, valproate induces CD20 expression and increases rituximab-induced CDC in a mouse model of B-cell lymphoma [7].

The anticonvulsant valproate was identified in 2001 as having inhibitory activity of class I and II HDACs [8] While valproate is the clinically most well characterised HDACi, and has been utilized in the treatment of epilepsy since the 1970s, several HDACis are shown to have effect on specific tumor types as single agent drugs, and hematological malignancies seem to be particularly sensitive to HDAC inhibitors. Accordingly, vorinostat (Zolinza^®^. or SAHA) and romidepsin (Istodax^®^) were approved by the Food and Drug Administration, USA, in 2006 and 2009, respectively, for the treatment of cutaneous T-cell lymphoma.

Chronic lymphocytic leukemia (CLL) is a heterogeneous disease with highly variable clinical outcome with survival varying from months to decades. Chemoimmunotherapy with fludarabine, cyclophosphamide and rituximab (FCR) has been the standard first-line therapy for younger patients with CLL, where addition of rituximab significantly improved treatment response [9]. For older patients who may not be able to tolerate FCR, the combinatorial treatment of chlorambucil with the second generation CD20 antibodies obinutuzumab or ofatumumab is now an option [10, 11]. However, although obinutuzumab and ofatumumab have induced longer lasting remissions than rituximab, relapse after treatment and CD20-antibody resistance is still a central issue in CLL. As compared to B-cell lymphomas and also to normal B-cells, CLL cells express lower levels of CD20 on their cell membrane, and the CDC response to anti-CD20 treatment has been shown to be related to the number of CD20 molecules on the cell surface [12]. Interestingly, the levels of CD20 on CLL cells have been shown to correlate to cytogenetic aberrations, in that trisomy 12 expresses the highest levels of CD20 while del11q, del13q and del17p all express comparable and low levels. Moreover, recent data show evidence for a NOTCH1 c7541_7542delCT mutation-driven epigenetic downregulation of CD20 expression. This downregulation is correlated to a worse response to rituximab-containing therapy in patients with NOTCH1 c7541_7542delCT mutation, but also to sensitivity to valproate-induced upregulation of CD20 in NOTCH1 c7541_7542delCT mutant cells during *in vitro* treatment of patient cells [9, 13].

The aim of the present study was to improve treatment with CD20 antibodies in CLL *in vivo* by induction of CD20. Therefore, three CLL patients were treated with the HDACi valproate according to the PREVAIL study (NCT02144623). All three treated patients were women with del13q and wild-type NOTCH1. In contrast to previous reports, and in spite of valproate-mediated induction of global histone acetylation, no upregulation of CD20 *in vivo* could be detected in these patients. To understand the molecular mechanisms for the unresponsiveness of CD20 induction to HDAC inhibition by valproate, we investigated the levels of the activating histone mark H3K9ac and the repressive histone mark H3K27me3 on the CD20 promoter in circulating lymphoma cells from patients and in the matched del13q/NOTCH1wt CLL cell line I83-E95. We found that in contrast to the CLL cell line I83-E95, in patients valproate transiently recruited the transcriptional repressor EZH2 and simultaneously induced the repressive histone mark H3K27me3 and the activating mark H3K9ac on the CD20 promoter in del13q/NOTCH1wt CLL *in vivo*, suggesting that valproate may induce a bivalent status of the CD20 promoter *in vivo*. Interestingly, valproate induced expression of EZH2 and global H3K27me3, suggesting that valproate may have transcriptionally repressive effects in CLL. Our results indicate new *in vivo* mechanisms of HDAC inhibitors, which may have clinical implications in future usage of HDACis.

## RESULTS

### Valproate induces CD20 expression and global acetylation of H3K9 in the CLL cell line I83-E95

**Figure 1:**
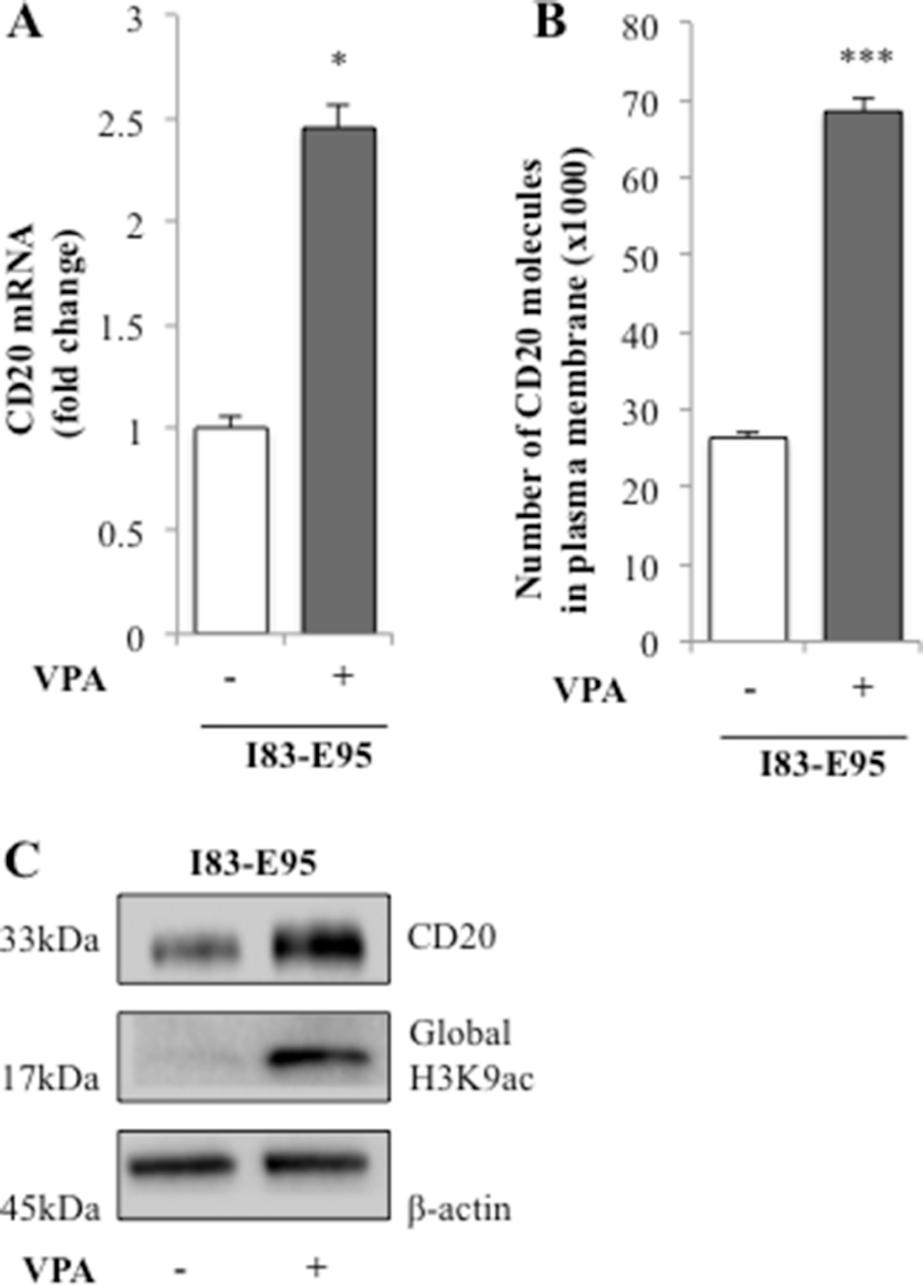
CD20 expression is induced by valproate in the I83-E95 CLL cell line I83-E95 cells were treated for 0h or 72h with valproate (VPA) at 1000 μM. CD20 expression was analysed by qPCR (**A**), FACS (**B**), and Western blot (**C**). (Mean values, bars ± s.e.m, *n* = 3). Global acetylation of H3K9 was assessed by Western blot (C) and β-actin was used as equal loading control. Stars indicate statistical significance (*p < 0.05; ***p < 0.001).

In the VALFRID study, we have previously shown that *in vivo* valproate treatment for 72 hours at pharmacologically relevant doses results in upregulation of CD20 mRNA and protein in lymphoma cells from DLBCL patients with concomitant acetylation of H3K9 in peripheral blood mononuclear cells [6]. To study whether valproate treatment could be a possible way to improve anti-CD20 treatment in CLL by upregulating CD20 expression, the CLL-cell line I83-E95 was initially used. I83-E95 cells carry del13q as reported by the supplier DSMZ. Since mutation of c7541_7542delCT, resulting in a frameshift deletion of NOTCH1, represents more than 80% of NOTCH1 mutations in CLL, and has been shown to affect CD20 expression in CLL [13], the NOTCH1 genomic region between 7270–7680 bp in I83-E95 cells was sequenced. According to our analysis, I83-E95 cells are wild type regarding NOTCH1 c7541_7542delCT. As shown in Figure 1, incubation with the pharmacologically relevant levels of 1000 μM of valproate for 72 hours induced expression of CD20 mRNA and protein as measured by qPCR (A), FACS (B) and Western blot (C) in I83-E95 cells. Moreover, as measured by Western blot, also global acetylation of H3K9 was increased (C), indicating that HDAC inhibition had occurred in response to valproate.

### Valproate at levels resulting in global acetylation of H3K9 does not increase CD20 expression in three CLL patients

To study the *in vivo* effects of valproate in CLL patients, the PREVAIL study was initiated (NCT02144623). In this study, CLL patients ages 18–80 years without treatment indication, received valproate orally three times daily at 60 mg/kg/day for three days in three 21-day cycles. On day 1–4 (i.e., hours 0, 24, 48 and 72) of each cycle, circulating leukemic cells from peripheral blood were isolated and serum-levels of valproate analysed. Four patients were included in the study. Three (patient 1, 2 and 4) were women aged 65, 77 and 71 years, respectively. Patient 3 was a 75-year old man, who had to be excluded because of a hearing disorder (Table 1). All included patients had del13q. No patients had del11q, trisomy 12 or del17p (Table 2). Sequencing for the NOTCH1 c.7541_7542 mutation showed wild type genotype in all patients. As shown in Table 3, valproate treatment resulted in serum concentrations around 1000 μM of valproate, which are comparable to serum levels in DLBCL patients from the VALFRID study, and also to what was used *in vitro* in I83-E95 cells. Patient 1 and patient 4 had to reduce the valproate dose due to p-valproate > 1200 μM and to fatigue and dizziness as instructed by the study protocol.

**Table 1:** Patient characteristics of the PREVAIL study

Patient number	Age	Sex	Study completion
01	65	Female	Completed study
02	77	Female	Completed study
03	75	Male	Excluded due to hearing impairment grade 3*
04	71	Female	Completed study

* Since a rare side-effect of valproate is hearing impairment, patients with impaired hearing were excluded from the study.

**Table 2:** FISH and NOTCH1 gene status of the CLL patients

Patient number	del13q	del11q	trisomy 12	del17p	NOTCH1 sequence
01	yes	nd	nd	nd	wt
02	yes	nd	nd	nd	wt
03	yes	nd	nd	nd	wt
04	yes	nd	nd	nd	wt

Del13q, del11q, trisomy 12 and del17 p cytogenetic aberrations were analysed by fluorescence *in situ* hybridization (FISH) and NOTCH1 mutation 7541_7542delCT was analysed as described in materials and methods. (nd: not detected).

**Table 3:** Administered valproate dose and plasma level of valproate

Pat no	Cycle	Valproate dose/kg/day	P-valproate (μM)			
			Day 1	Day 2	Day 3	Day 4
01	1	60	< 20	1108	1505*	611
	2	60	< 20	1046	1568*	502
	3	30	< 20	605	796	889
02	1	60	< 20	1198	781	826
	2	60	< 20	669	798	985
	3	60	< 20	461	833	Not done
04	1	60	< 20	685	932	1123
	2	60	< 20	908	1288*	638
	3	30	< 20	638	852	833

Patients were administered oral valproate divided on three doses per day, starting after blood sampling morning day 1. Last dose was given evening day 3. Cycle length was 21 days. All sampling was performed in the morning. *Day 3 doses 2 and 3 were excluded during due to fatigue and dizziness.

**Figure 2:**
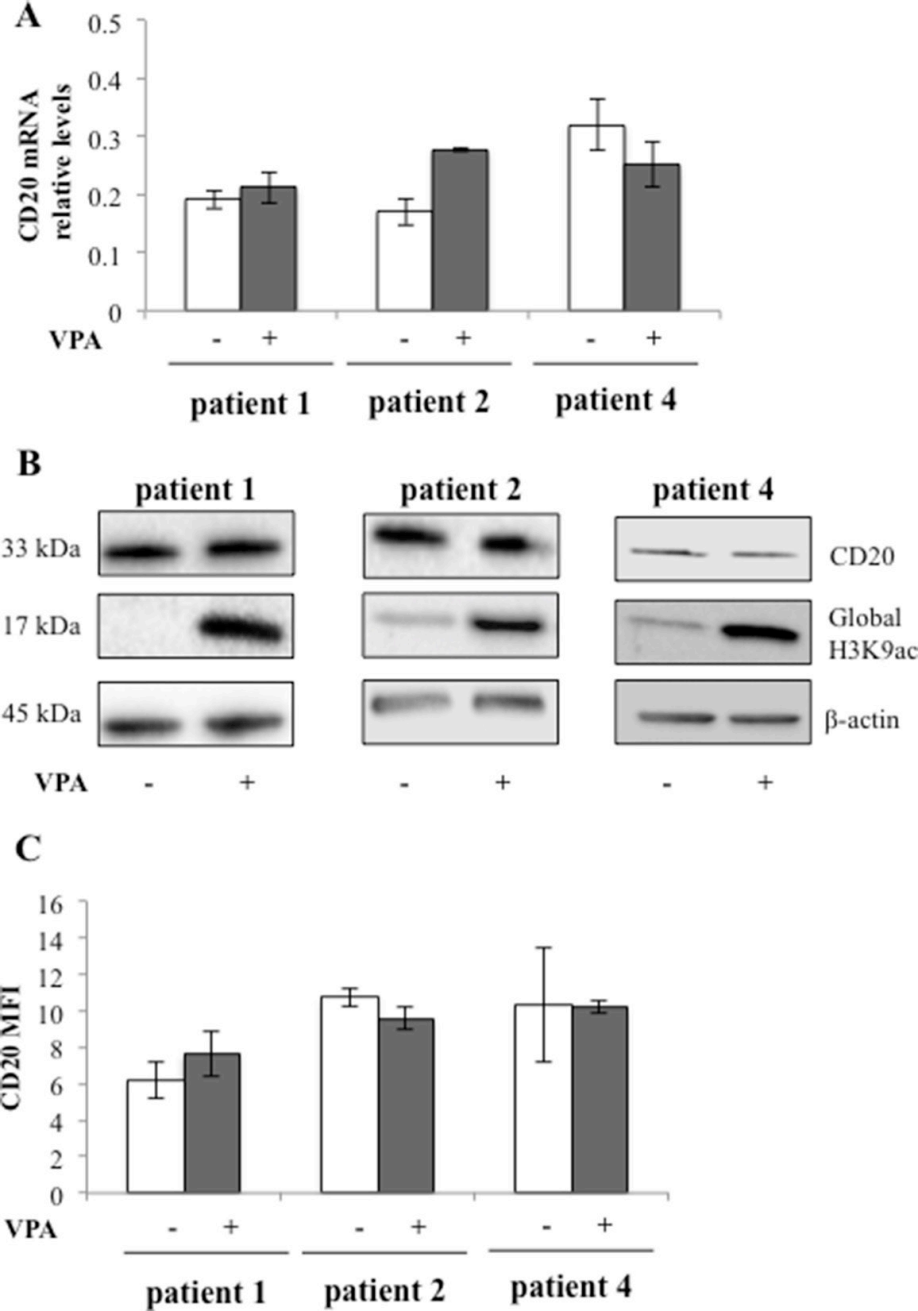
CD20 expression in patient cells is not affected by valproate despite induction of global H3K9 acetylation Circulating previously untreated CLL cells from three patients were cultured *in vitro* for 48 h with or without 1000 μM valproate (VPA). CD20 mRNA levels were analysed by qPCR (**A**). CD20 protein and H3K9 acetylation levels were analysed by Western blot (**B**). β-actin was used as equal loading control. Plasma membrane levels of CD20 were analysed by FACS (**C**). Bars show range of two experiments. MFI = mean fluorescence intensity.

To study whether valproate could induce CD20 expression in patient del13q/NOTCH1wt CLL cells, and to ascertain that valproate treatment at a clinically relevant dose could result in HDAC inhibition, untreated leukemic cells from patient 1, 2 and 4 were incubated with 1000 μM of valproate *in vitro*. After 72 hours CD20 mRNA levels were analysed by qPCR (Figure 2A) and CD20 protein levels were analysed by Western Blot and flow cytometry (Figure 2B and 2C). Moreover, the global H3K9 acetylation was addressed. As expected, valproate treatment resulted in global H3K9 acetylation in the patient cells (Figure 2B). However, in contrast to the results from the I83-E95 cell line, no upregulation of CD20 mRNA or protein could be detected in cells from patients 1, 2 and 4 treated *in vitro*.

**Figure 3:**
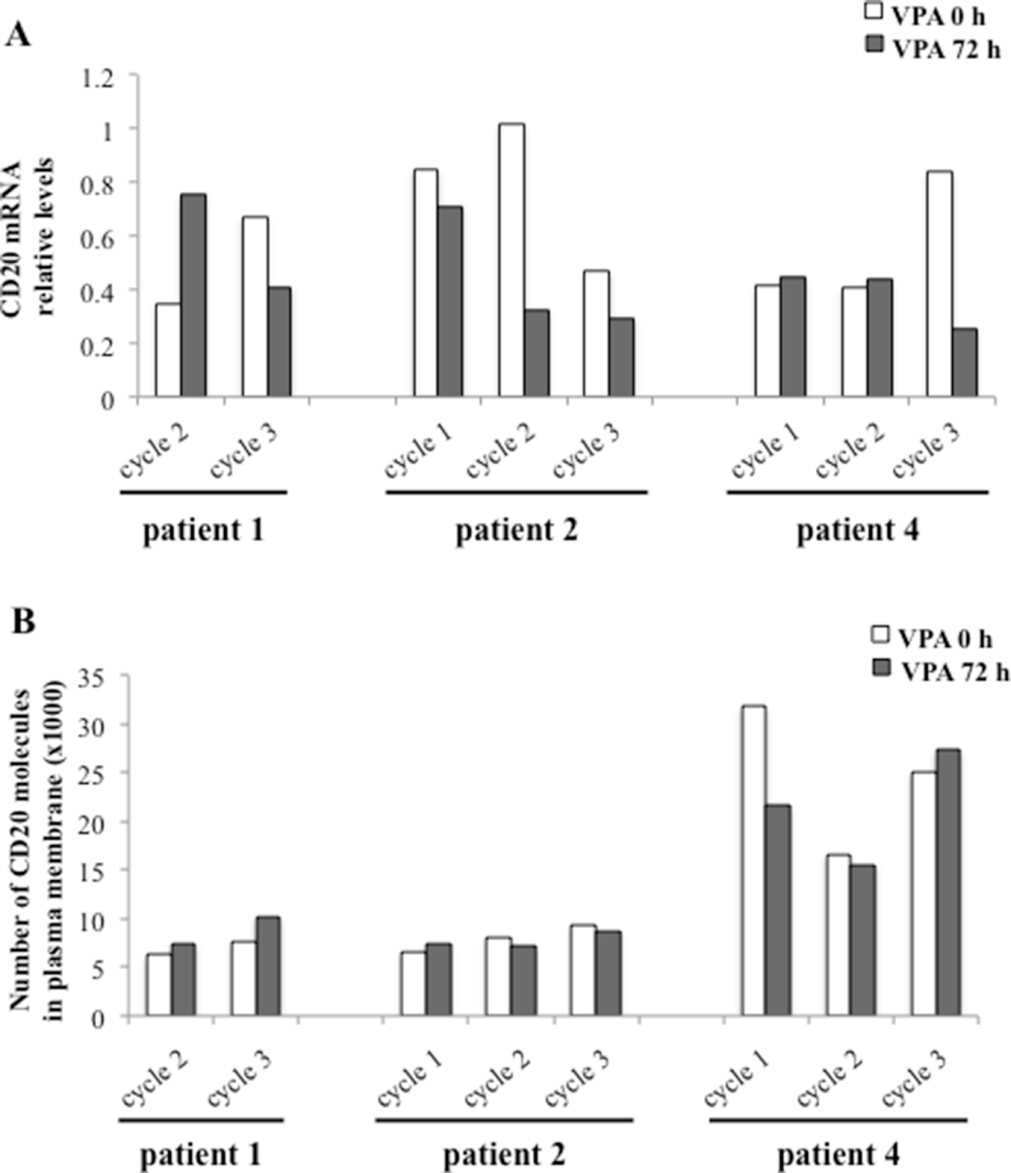
Repeated valproate-treatment *in vivo* does not induce expression of CD20 mRNA or surface protein in three CLL patients Three CLL patients were treated with valproate (VPA) for three days in three 21-day cycles. For serum levels of valproate, please see Table 3. After 0 and 72 hours of valproate treatment in each cycle, levels of CD20 mRNA (**A**) and membrane protein (**B**) were analysed by qPCR and flow cytometry, respectively. Quantibrite beads were used to allow the comparison of the number of CD20 molecules in the plasma membrane between different treatment cycles.

We next wanted to analyse whether *in vivo* treatment with valproate of chronic lymphocytic leukaemia patients could affect CD20 expression, and also whether repeated exposure to valproate could affect the levels of CD20. To that end, CD20 expression of circulating leukemic cells from patient 1, 2 and 4 sampled *in vivo* during cycle 1, 2 and 3 of the PREVAIL study was analysed with qPCR (Figure 3A) and flow cytometry (Figure 3B). To allow the comparison of the number of CD20 molecules in the plasma membrane between treatment cycles, Quantibrite beads were utilised. However, no consistent valproate-related effects on expression of CD20 mRNA or surface protein could be detected in the patient samples regardless of treatment cycle, suggesting that valproate does not upregulate CD20 in del13q/NOTCHwt CLL *in vivo*.

### Valproate mediates simultaneous induction of the repressive histone mark H3K27me3 and the activating histone mark H3K9ac on the CD20 promoter in CLL patients *in vivo*

**Figure 4:**
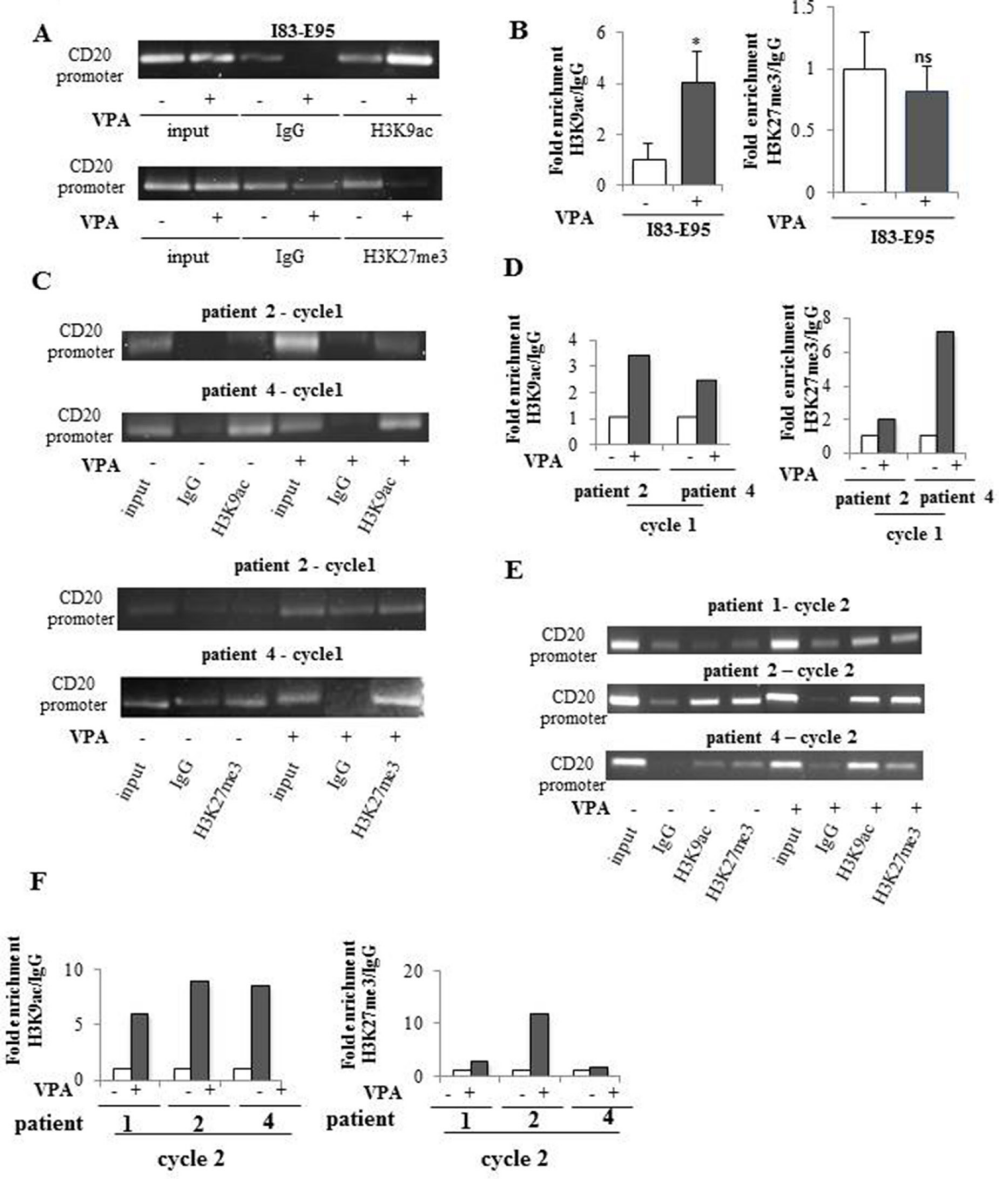
Valproate induces simultaneous H3K9ac and H3K27me3 in the CD20 promoter in CLL patients *in vivo* but not in the I83-E95 CLL cell line I83-E95 cells were incubated with or without valproate (VPA) at 1000 μM for 72 hours and subjected to ChIP with anti-H3K9ac (**A**, top) or anti-H3K27me3 (A, bottom) antibodies. Patient samples collected after 0 and 72 hours of valproate-treatment from cycle 1 (**C**) and cycle 2 (**E**) of the PREVAIL study were subjected to ChIP with anti-H3K9ac and anti-H3K27me3 antibodies. Levels of H3K9ac and H3K27me3 on the CD20 promoter were evaluated by densitometry analysis and expressed in graphs (**B**, **D**, **F**). (Mean values, bars ± s.e.m, *n* = 3). Stars indicate statistical significance (*p < 0,05; ns: not significant). Due to sample limitation, experiments C-F were performed once.

We next wanted to understand why valproate did not induce expression of CD20 mRNA and protein in CLL patients, while it did in the CLL cell line I83-E95. Since several studies suggest that CD20 expression is epigenetically regulated (3–6), valproate-related effects on activating and repressive histone marks on the CD20 promoter were compared in the I83-E95 cell line and in patient samples. To that end, the I83-E95 cell line was incubated with valproate at 1000 μM for 72 hours, and patient samples were collected after 0 and 72 hours of valproate-treatment from cycle 1 and 2 of the PREVAIL study. ChIP was performed with antibodies against the activating histone mark H3K9ac, or the repressive histone mark H3K27me3 after which the CD20 promoter in the sampled material was analysed. As shown in Figure 4, valproate induced increased acetylation of H3K9 of the CD20 promoter both in the I83-E95 cell line (A, B) and in patient cells (C, D). This is consistent with the valproate-mediated induction of CD20 expression in the I83-E95 cell line, but it does not explain why CD20 is not induced in circulating leukemic cells from patients. However, interestingly, although valproate did not affect the repressive histone mark H3K27me3 in the I83-E95 cell line (A, B), a valproate-induced increase in H3K27me3 in the CD20 promoter of all three patient cells was observed, both in valproate naïve patients, previously unexposed to valproate (patients 2 and 4; cycle1; Figure 4C, 4D), and valproate exposed patients (patient 1, 2 and 4, cycle 2; Figure 4E, 4F). It was not possible to analyse H3K9ac and H3K27me3 of the CD20 promoter in patient 1 cycle 1 due to lack of material. This suggests that the resistance to valproate-induced expression of CD20 in CLL *in vivo* could be dependent on simultaneous induction of H3K27me3 and H3K9ac on the CD20 promoter.

### Valproate simultaneously increases expression of the transcriptional repressor EZH2 and global H3K27 trimethylation in patient CLL cells

**Figure 5:**
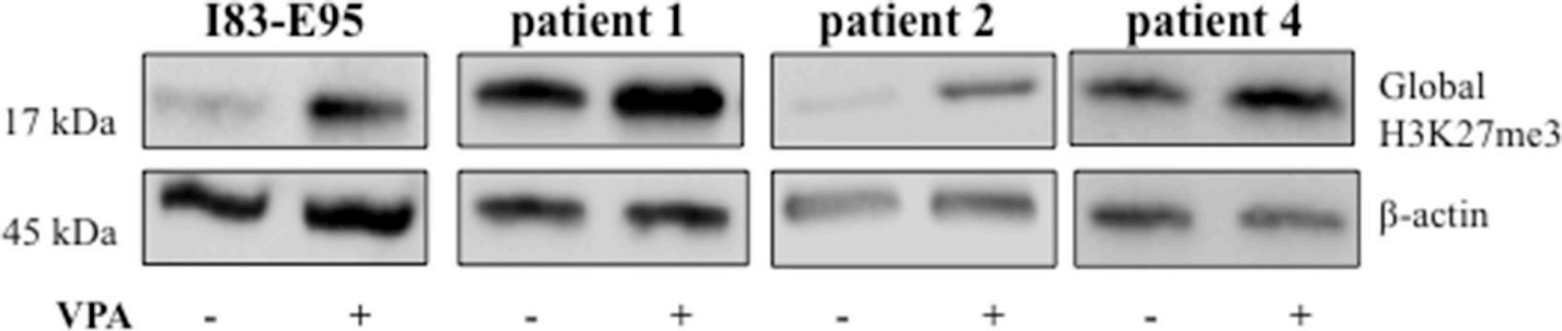
Global H3K27me3 is increased by valproate in CLL patients and in the I83-E95 CLL cell line I83-E95 cells and circulating untreated CLL cells from patients were incubated with or without 1000 μM of valproate. After 48 hours total protein extracts were prepared and global H3K27me3 was analysed by Western blot. β-actin was used as equal loading control. One out of two independent experiments is shown. For experiment number 2, please see Supplementary Figure 1.

**Figure 6:**
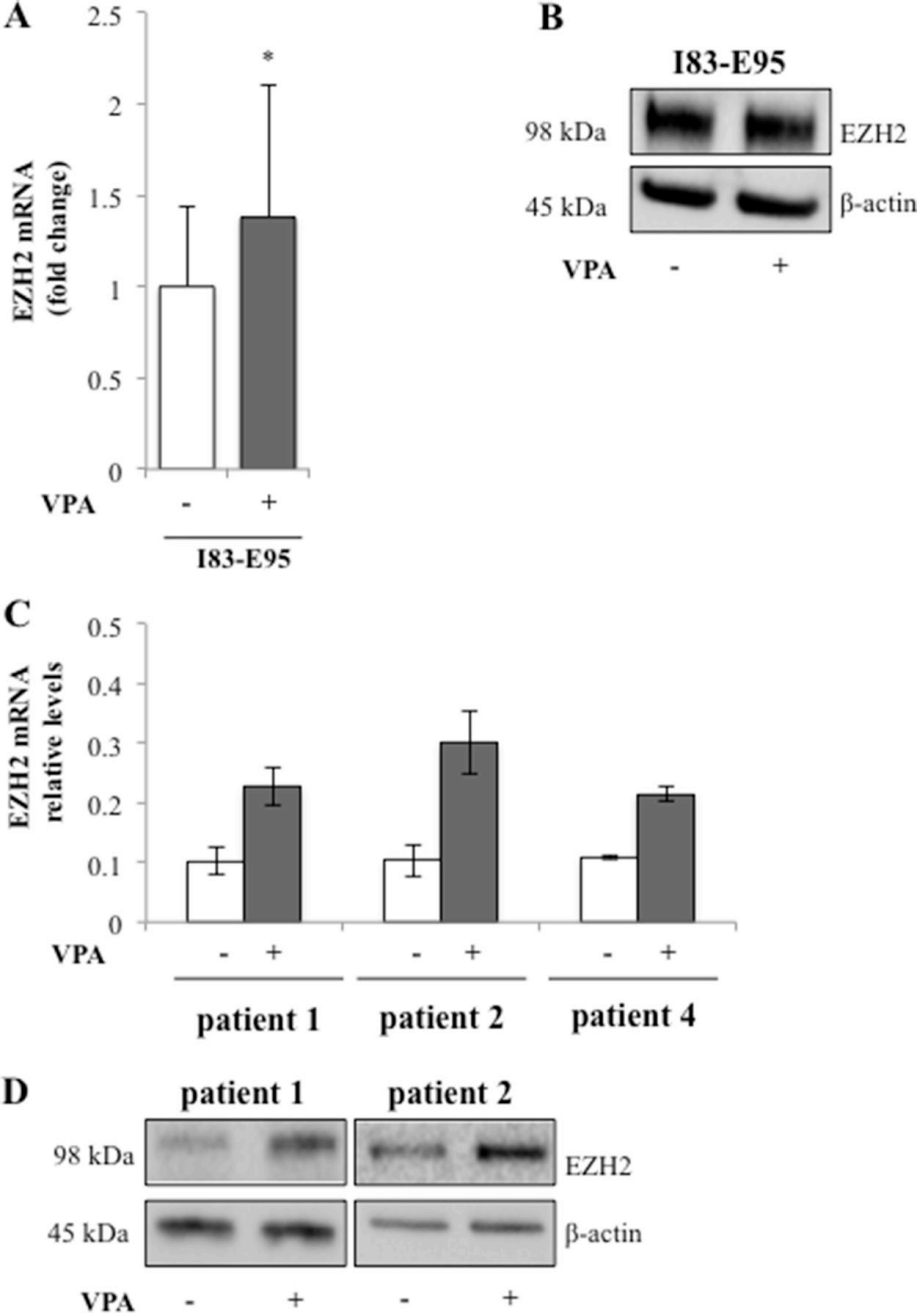
Valproate induces expression of the transcriptional repressor EZH2 in CLL patients I83-E95 cells and circulating untreated CLL cells from patients were incubated with or without 1000 μM of valproate. After 48 hours’ levels of EZH2 mRNA in I83-E95 cells (**A**, mean values, bars ± s.e.m, *n* = 4, *p < 0,05) and patient cells (**C**, mean values, bars show range of two experiments, *n* = 2) and protein (**B**, **D**) were analysed by qPCR and Western blot respectively. One out of two independent Western blots is shown. For Western blot number 2, please see Supplementary Figure 1B.

To study whether the valproate-induced H3K27me3 in patient cells was isolated to the CD20 promoter or a general phenomenon, previously untreated patient cells from patient 1, 2, and 4 and the I83-E95 cell line were cultured *in vitro* with or without valproate at 1000 μM for 48 hours, after which global H3K27me3 was analysed by Western blot. To our surprise, valproate induced global H3K27me3 both in patient cells, and in the I83-E95 cell line (Figure 5 and Supplementary Figure 1). To search for a molecular mechanism explaining the global H3K27me3 induced by valproate, levels of EZH2 were analysed. EZH2 is the catalytic subunit of the polycomb repressor complex 2 (PRC2) where it exerts trimethylation of H3K27, resulting in repression of gene expression (18). To assess whether valproate could induce EZH2 expression in CLL, possibly resulting in global H3K27me3, I83-E95 cells and untreated leukemic cells from patient 1, 2 and 4 were incubated with or without 1000 μM of valproate *in vitro*. After 48 hours, levels of EZH2 mRNA and protein were analysed by qPCR (Figure 6A and 6C) and Western Blot (Figure 6B and 6D). Due to lack of material, no Western blot could be performed in patient 4. Valproate treatment induced a modest but statistically significant increase of EZH2 mRNA in I83-E95 cells, while no obvious effects on EZH2 protein could be demonstrated. Nevertheless, a clear increase of EZH2 mRNA and protein in response to valproate-treatment was seen in the patients studied.

In conclusion, although no causal connection can be shown, our data suggest that valproate may increase global H3K27me3 by induction of EZH2 expression in del13q/NOTCH1wt CLL patients.

### Valproate transiently recruits the transcriptional repressor EZH2 to the CD20 promoter in CLL *in vivo* but not in I83-E95 cells

**Figure 7:**
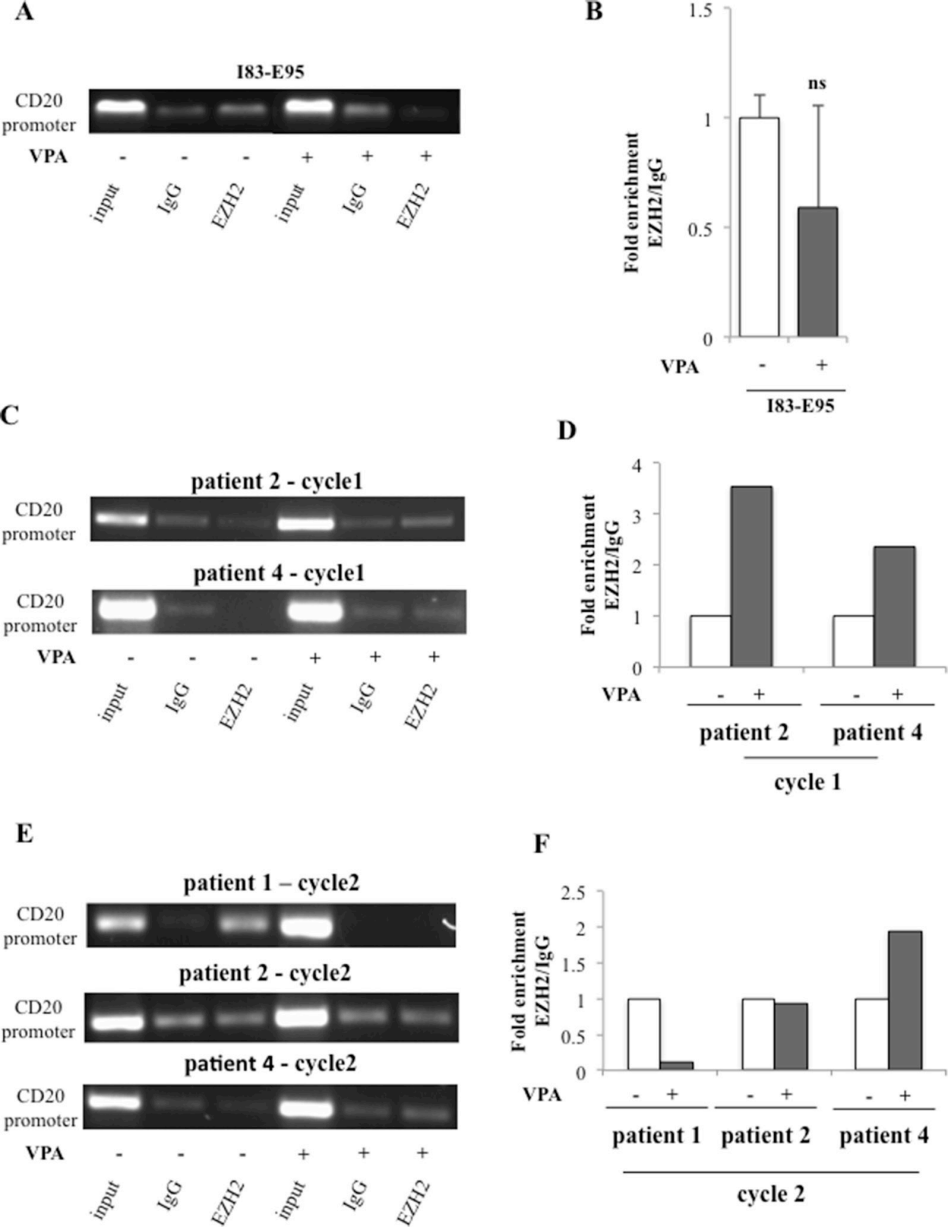
Valproate transiently recruits the transcriptional repressor EZH2 to the CD20 promoter *in vivo* but not *in vitro* I83-E95 cells were incubated with or without valproate (VPA) at 1000 μM for 72 hours and subjected to ChIP with anti-EZH2 antibody (**A**). Patient samples collected after 0 and 72 hours of valproate-treatment from cycle 1 (**C**) and cycle 2 (**E**) of the PREVAIL study were subjected to ChIP with anti-EZH2 antibody. Levels of EZH2 on the CD20 promoter were evaluated by densitometry analysis and expressed in graphs (**B**, **D**, **F**). (Mean values, bars ± s.e.m, *n* = 3; ns: not significant). Due to sample limitation, experiments C–F were performed once.

To study whether the valproate-mediated induction of EZH2 expression and increase of H3K27me3 on the CD20 promoter was connected to recruitment of EZH2, the binding of EZH2 to the CD20 promoter in the I83-E95 cell line and in *in vivo* treated patient cells was analysed by ChIP. For this purpose, I83-E95 cells were incubated with or without valproate at 1000 μM *in vitro* for 72 hours. Analysed patient samples were collected after 0 and 72 hours of valproate-treatment from cycle 1 and 2 of the PREVAIL study. As expected (Figure 7A, 7B), no enrichment of EZH2 binding to the CD20 promoter was seen in I83-E95 cells, consistent with the lack of induction of H3K27me3 on the CD20 promoter and also to the increased CD20 expression in response to valproate in these cells. However, interestingly, enrichment of EZH2 on the CD20 promoter was induced by valproate in treatment naive cells from patient 2 and patient 4 in cycle 1 (Figure 7C, 7D). It was not possible to analyse binding of EZH2 to the CD20 promoter in patient 1 cycle 1 due to lack of material. Valproate-induced enrichment of EZH2 on the CD20 promoter was not evident in patient cells repeatedly exposed to valproate (patient 1, 2 and 4 cycle 2, Figure 7E, 7F). In summary, these data may suggest that valproate recruits EZH2 to the CD20 promoter in del13q/NOTCHwt CLL patients during initial treatment, but that this effect is lost upon repeated valproate-exposure.

## DISCUSSION

Chronic lymphocytic leukaemia is a disease of the elderly, with a median age of 70 years at disease debut. Hence, many CLL patients cannot tolerate heavy chemotherapy, and milder treatment options are necessary. In this context, the limited side-effects connected to treatment with CD20-antibodies, either alone or as combination-therapy, has proven advantageous. During recent years protein kinase inhibitors such as ibrutinib and idelalisib have been introduced to treatment also of elderly patients with promising results [14]. However, both ibrutinib and idelalisib inhibit immune-cell mediated mechanisms of CD20 antibodies, and are also shown to downregulate CD20 expression in primary CLL cells [15]. Therefore, new strategies to improve anti-CD20 treatment in the era of protein kinase inhibitors are in the spotlight.

It has for long been known that CLL cells express lower levels of CD20 on their cell surface than normal B-cells, and also lower than follicular lymphomas, which are commonly successfully targeted with anti-CD20 treatment. Although the reason for this it not known, epigenetic downregulation of CD20 expression by an increased presence of HDACs at the CD20 promoter has been shown in NOTCH1mutated CLL [13]. Moreover, regulation of CD20 expression by histone acetylation of the CD20 promoter has been shown in several cases of B-cell malignancies [5, 7]. In addition, rituximab-resistant B-cell lymphoma cell lines may down-regulate CD20 expression by recruitment of HDACs and reduced CD20 promoter activity [4, 5].

It has previously been shown that HDAC inhibition by valproate requires 48 hours to induce chromatin decondensation and thus allow for maximal transcription [16, 17]. Moreover, valproate treatment results in a robust increase of CD20 expression within 48 hours in eight B-cell lymphoma cell lines [7], and our data show that valproate induces maximal CD20 mRNA and protein upregulation in the CLL cell line I83-E95 after 48 hours (Supplementary Figure 2). Additionally, CD20 expression is increased in diffuse large B-cell lymphoma patients after 48 hours of valproate treatment [6]. Therefore, to assess whether anti-CD20 treatment of CLL could be improved by a valproate-mediated increase of CD20 on CLL cells, the PREVAIL study was initiated. The study was designed to allow chromatin decondensation and CD20 mRNA transcription during a 72-hour treatment period of valproate. Also, since valproate has been shown to induce long-term effects on the epigenome through modulation of DNA methylation [16, 18], three treatment cycles with 21-day interval were administered to study potential long-term effects of valproate on CD20 expression. Indeed, we did observe valproate-induced global acetylation of H3K9 in patient lymphoma cells after 72 hours, indicative of sufficient treatment time for required HDAC inhibitory effects. However, although expression of CD20 mRNA seems to be more sensitive to valproate treatment than CD20 protein levels on the cell surface, no consistent effects on the expression of CD20 mRNA or protein could be observed, regardless of treatment cycle. It cannot be excluded that the varying levels of CD20 mRNA depend on alternative splice variants, as previously described in CLL [19]. Our data are in contrast to data from Pozzo *et al* [13], where *in vitro* treatment with valproate could upregulate CD20 expression both in primary CLL cells and in the CLL MEC-1 cell line, regardless of NOTCH1 mutation. However, Pozzo *et al* observe the highest levels of CD20 in patients with trisomy 12, while all patients included in the present study had del13q, which is linked to a lower expression of CD20. Moreover, the dose of valproate utilised in the study by Pozzo et al (3000 μM) is above a clinically relevant dose. Interestingly, Pozzo *et al* show a link between NOTCH1 c7541_7542delCT mutation and downregulation of CD20. However, the patients included in the present study were wild type regarding c7541_7542delCT mutation, excluding that this mutation contributed to the lack of valproate-mediated upregulation of CD20.

To understand why valproate could upregulate CD20 in the del13q/NOTCHwt CLL cell line I83-E95, but not in CLL del13q/NOTCH1wt patients, we investigated the pattern of activating and repressive histone marks on the CD20 promoter in the I83-E95 cell line, and in three CLL patients. We show that although valproate induces acetylation of the activating histone mark H3K9 on the CD20 promoter both in patients and in cell line, it increases the repressive histone mark H3K27me3 on the CD20 promoter in patients but not in the cell line, and this increase persists after repeated valproate-exposure. The differing regulation of CD20 expression between the I83-E95 cell line and the patient material is intriguing. Although the genotype of the cell line was matched to the patient material with regard to del13q and NOTCH1 wild type, H3K27me3 was induced by valproate on the CD20 promoter in patients but not in the cell line. In line with this differing CD20 promoter status in patients *vs* cell line, the transcriptional repressor EZH2 was transiently recruited to the CD20 promoter in patients, but not in the cell line. We cannot explain this finding, but believe that it emphasizes the importance of conducting research *in vivo*, in contrast to immortalized cell lines.

The bivalent status of the CD20 promoter in response to valproate is in our opinion an important discovery. CD20 levels are known to fall during terminal differentiation of activated B-cells into plasma cells or memory B-cells [1]. While most previous studies showing valproate-induced upregulation of CD20 have been performed in Burkitt lymphoma and DLBCL cells, emanating from germinal centre B-cells, [4, 6, 7], CLL cells correspond to memory B-cells from late stages of B-cell differentiation. An increasing number of studies suggest that B-cell differentiation is epigenetically regulated by histone modifications [20, 21]. Hence, since valproate has been shown to possess differentiation inducing properties [22], it is possible that differentiation modulation by valproate in the epigenetic landscape of CLL contributes to the H3K27me3 of the CD20 promoter in patients. This is of particular interest in the light of the observed global trimethylation of H3K27 in both patients and cell line. It seems as if valproate can induce a repressive chromatin status in CLL, and we speculate that this is coupled to the differentiation inducing effects of valproate.

Overexpression of the transcriptional repressor EZH2 is implicated in tumorigenesis and correlates with poor prognosis in several tumour types [23–27]. In B-cell lymphomas, heterozygous gain-of-function mutations of residues within the catalytic SET domain of EZH2 occur in diffuse large B-cell lymphoma (DLBCL) and follicular lymphoma, and result in enhanced H3K27 trimethylation and transcriptional repression [28]. In CLL, EZH2 has been shown to be a cellular prosurvival factor in clinically aggressive cases, and suggested as a potential therapeutic target [29].

Interestingly, valproate-treatment induced expression of EZH2 in the studied patients, possibly explaining the induction of global H3K27me3. However, although valproate induced global H3K27me3 in the I83-E95 cell line, it did not induce expression of EZH2 protein, suggesting that the effects of valproate on global H3K27me3 could be regulated also by other mechanisms.

Moreover, although the material is small, we observe valproate-induced recruitment of EZH2 to the CD20 promoter in valproate-naïve patient cells. The effect is not evident in patients repeatedly exposed to valproate, perhaps dependent on late epigenetic effects induced by initial valproate exposure. Although it cannot be excluded that the increased EZH2 levels induced by valproate influences EZH2 recruitment, the distinct mechanisms transiently recruiting EZH2 to the CD20 promoter *in vivo*, but not in the cell line, remain to be investigated.

In summary, consistent with previous results from us and from others (5, 6), we show that valproate has expected effects on the H3K9 acetylation of the CD20 promoter. However, in contrast to earlier findings, our data suggest that in del13q/NOTCH1wt CLL *in vivo*, valproate also transiently recruits EZH2 to the CD20 promoter corresponding to H3K27trimethylation and resistance to valproate-induced upregulation of CD20. Moreover, valproate-treatment simultaneously induces the transcriptional repressor EZH2 and global trimethylation of H3K27. To our knowledge, the ability of HDACis to induce repressive histone marks or to induce or recruit transcriptional repressors has not previously been shown in malignant cells, neither *in vivo* nor *in vitro*. We believe that our findings shed new light on the function of HDACis in CLL, and future studies on combination therapy with HDACis and EZH2 inhibitors in B-cell malignancies are warranted.

## MATERIALS AND METHODS

### Eligibility

Please see ClinicalTrials.gov: ID NCT02144623 for eligibility for the PREVAIL (Pre-treatment with Valproate before Immunotherapy targeting CD20 in CLL) study. Inclusion criteria were age 18–80 years, histologically confirmed CLL according to the WHO classification, and total white blood cell count > 20 × 10^9^/L. Patients with a treatment indication for CLL were excluded from the study, and since valproate has been shown to cause a hearing impairment in rare cases, also all patients with a hearing impairment more than grade 2 according to the CTCAE v4.0 were excluded from the study. Informed consent was obtained from patients in accordance with good clinical practice and federal and institutional guidelines governing registered clinical trials in accordance with the declaration of Helsinki. The study was approved by the Internal Review Board of the Regional Ethical Committee of Lund (Number 2014/215). CLL cases were characterized by fluorescence *in situ* hybridization (FISH) of the cytogenetic abnormalities del11q, trisomy 12, del13q and del17p by the Department of Clinical Genetics, Skåne University Hospital.

### Study treatment

Valproate (Ergenyl^®^ or Orfiril^®^) was administered orally every 8 hours for three days in three 21-day cycles at a starting dose of 60 mg/kg/day. In some instances, the dose was reduced to 30 mg/kg/day, as indicated in Table 3.

### Pharmacokinetics

Levels of total plasma valproate were measured before morning dose of valproate day 1, 2, 3 and 4 by a homogenous enzyme-immunoanalysis technique at the Department of Clinical Chemistry at Skåne University Hospital.

### Cell culture

The I83-E95 chronic lymphocytic leukemia cell line was obtained from the Leibniz Institute DSMZ - German collection of Microorganisms and Cell culture and was passaged for less than six months after receipt. According to DSMZ, I83-E95 cells carry del13q and are immortalized by EBV infection. Short tandem repeat analysis on DNA was performed by DMSZ. I83-E95 cells were cultured in Iscove's Modified Dulbecco's Medium (IMDM) from Gibco, Life Technologies, USA), supplemented with 20% fetal calf serum at a concentration of 300.000 cells/ml at 37^°^C in 5% CO_2_.

Circulating lymphoma B-cells were isolated from the peripheral blood of CLL patients 0, 24, 48 and 72 hours after start of valproate treatment using the MACSxpress B-CLL cell isolation kit according to the manufacturer´s instruction (cat.no. 130-104-445, Miltenyi Biotec, Germany). After the cell separation, remaining erythrocytes were lysed with MACSxpress Erytrocytes depletion kit (cat. no. 130-098-196 Miltenyi Biotec, Germany) according to the manufacturer´s instruction. CLL cells from patients were cultured under the same conditions as the cell line.

Cultured cells were treated with valproate (Sigma Aldrich, USA) at the concentration of 1000 μM.

### Flow cytometry

The surface CD20 and CD19 expression was detected using the following antibodies: mouse monoclonal anti-human CD20 (L27) PE (cat. no. 347201, BD Pharmingen) and mouse monoclonal anti-human CD19 APC-Cy7 (cat. no. 557791 BD Pharmingen), along with the corresponding isotype-matched controls (PE mouse IgG1_Κ_, BD and APC-cy7 mouse IgG1 _Κ_, Biolegend), using a FACS Canto (BD Biosciences, U.S.A) flow cytometer. During the clinical trial, CD20 expression between cycles was analysed utilizing the PE Quantibrite beads (BD Biosciences, U.S.A) allowing calculation of the number of CD20 molecules in the plasmamembrane (expressed as thousands per cell) using the Excel formula provided by BD Biosciences. In *in vitro* experiments on patient cells, median fluorescence intensity was utilized to estimate valproate-induced changes in CD20 expression.

### Real time-PCR

Total RNA was isolated using the RNeasy Mini Kit (cat. No. 74104 Qiagen, Hilden Germany) according to the manufacturer's instructions. RNA was reversely transcribed using High-Capacity cDNA Reverse Transcription Kit (cat. no. 4387406 Applied Biosystems Inc., Foster City, CA, U.S.A.) with random hexamer primers according to the manufacturer's instructions. Quantitative PCR (qPCR) was carried out using TaqMan probe-based chemistry (Applied Biosystems) using the probes for CD20 (Hs00544819_m1 MS4A1), GAPDH (Hs02758991_g1 GAPDH) and β_2_-Microglobulin (Hs00984230_m1 β_2_MG ). The amplification reactions were all performed in triplicates in a StepOnePlus machine (Applied Biosystems). Data were collected and analysed using the Applied Biosystems StepOneTMReal-Time PCR Software v2.0. The relative quantification in gene expression was determined using the ΔΔCt method.

### Western blot

Patient cells and I83-E95 cells were lysed in RIPA buffer (50mM Tris-HCl pH 7.8, 150 mM NaCl, 1 mM EDTA, 1% Nonidet P-40). Samples for immunoblotting were prepared using Laemmli buffer (cat. no. 161-0747, Bio-rad). Proteins were separated by SDS–PAGE (4561093, 4–20% Mini-Protean TGX gel, Bio-Rad) and transferred to a Hybond ECL membrane (GE Healthcare, Uppsala, Sweden). Primary antibodies used were: polyclonal mouse anti-CD20 (ab88247, Abcam), monoclonal mouse anti-β-actin (A2228, Sigma A), pAb rabbit H3K9ac (cat. no. 07-352 Merck Millipore, Darmstadt Germany), mAb rabbit tri-methyl-Histone (K27) (C36B11) (9733P) and mAb rabbit EZH2 (D2C9) (cat. no. 5246P) both from Cell Signalling. The ECL kit (Biological Industries, Kibbutz Beit Haemek, Israel) was used to detect protein bands with a ChemiDoc TM XRS+ system (Bio-Rad).

### Chromatin Immunoprecipitation

ChIP experiments were performed using the EZ-Chromatin immunoprecipitation kit (cat. no. 17-371, Merck Millipore, Darmstadt, Germany) according to the manufacturer's protocol. Chromatin was cross-linked with 1% of formaldehyde (Sigma Aldrich, USA) for 10 minutes in room temperature and the reaction was stopped adding glycine at 0.125 mM. After two washes in cold phosphate-buffered saline (Gibco, USA), cells were lysed in SDS-lysis buffer provided by the manufacturer (100 μL × 1e6 cells). Chromatin sonication was performed in a Bioruptor PICO (Diagenode). Different settings were used in order to obtain fragments between 200–500 bps: I83–95 cells were subjected to 11 sonications (1 min each: 30” ON -30” OFF) and patient B-CLL cells were subjected to 7 sonications (1 min each: 30” ON -30” OFF). The antibodies utilized were: rabbit polyclonal anti-H3K9ac (cat. no. 07-352, Merck Millipore), monoclonal mouse anti-H3K27me3 (cat. no. 6002 Abcam, Cambridge UK), (1μg of each for 1e6 cells) and monoclonal mouse EZH2, clone AC22 (cat. no. 17-662, Merck Millipore) (2 μg for 4e6 cells). Normal rabbit IgG isotype was used as background control of immunoprecipitation. ChIP samples were analysed by semi-quantitative PCR using 3 μl of input control (1:100 in accordance with the manufacturer's protocol) and 4,5 μl of immunoprecipitated material. Specific primers were used to amplify the CD20 promoter in the region included between −948 and −659 bps: 5′- tgcctgccatatttcatccc-3′

(forward) and 5′-cccttgtgtcccctctcttt-3′(reverse). Quantification of PCR bands was performed using ImageLab software and fold enrichment of the immunoprecipitated DNA compared with the negative control IgG was calculated.

### NOTCH1 sequencing

Genomic DNA was extracted by All prep universal DNA/RNA/miRNA kit (cat.no. 80224, Qiagen) from the I83-E95 cell line and from patients 1, 2 and 4. The presence of c.7541_7542delCT in the exon 34 of the NOTCH1 gene was addressed. PCR products were obtained using the NEBNext Ultra II Q5 Master Mix (cat.no. M0544S, New England Biolabs) and specific primers 5′-agatg atgagctaccagggc-3′ (forward), 5′- aaagtttctacctggggcca-3′ (reverse) according to the manufacturer's instruction.

Purified PCR products were sequenced directly from both strands (Sanger sequencing was performed by the service of Eurofins^®^ (www.eurofins.de), with the following primers: 5′-cagcaaacatccagcagca-3′ (forward) and 5′-gagacgttggaatgcggg-3′(reverse) covering the region between 7270–7680 bp. The obtained sequences were aligned with the corresponding germline sequence (ref. seq. NM 017617.2), using the BLAST tool.

### Statistical analysis

Statistical analysis was performed using the paired two-tailed *t*-test. Stars represent conventional significance levels; single star indicates *p* ≤ 0.05, double stars *p* < 0.01, triple stars *p* < 0.001. Standard error of the mean (s.e.m) was calculated when the number of samples was three or more. Due to sample limitation, experiments were sometimes performed once or twice.

## SUPPLEMENTARY MATERIALS FIGURES


