# Supplementary material for: The HDAC inhibitor valproate induces a bivalent status of the CD20 promoter in CLL patients suggesting distinct epigenetic regulation of CD20 expression in CLL in vivo
Source: Oncotarget. 2017 Apr 8;8(23):37409–22. doi: 10.18632/oncotarget.16964 (PMC5514918; doi:10.18632/oncotarget.16964)
Supplement: Supplementary file 1 [file oncotarget-08-37409-s001.pdf]

The HDAC inhibitor valproate induces a bivalent status of the CD20 promoter in CLL patients suggesting distinct epigenetic regulation of CD20 expression in CLL *in vivo*

Supplementary Materials

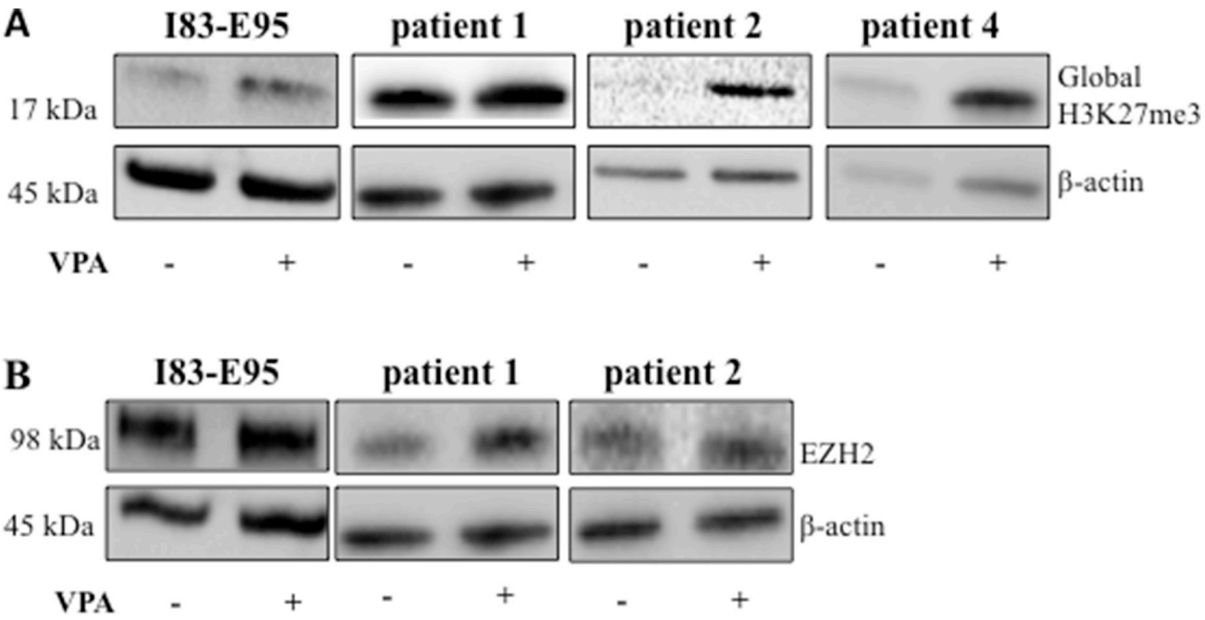

Supplementary Figure 1: Global H3K27me3 and EZH2 protein is increased by valproate in CLL patients. I83-E95 cells and circulating untreated CLL cells from patients were incubated with or without 1000 µM of valproate. After 48 hours' total protein extracts were prepared and global H3K27me3 (**A**) and levels of EZH2 (**B**) protein were analysed by Western blot. One out of two independent experiments is shown. For experiment number 1, please see Figure 5 and Figure 6.

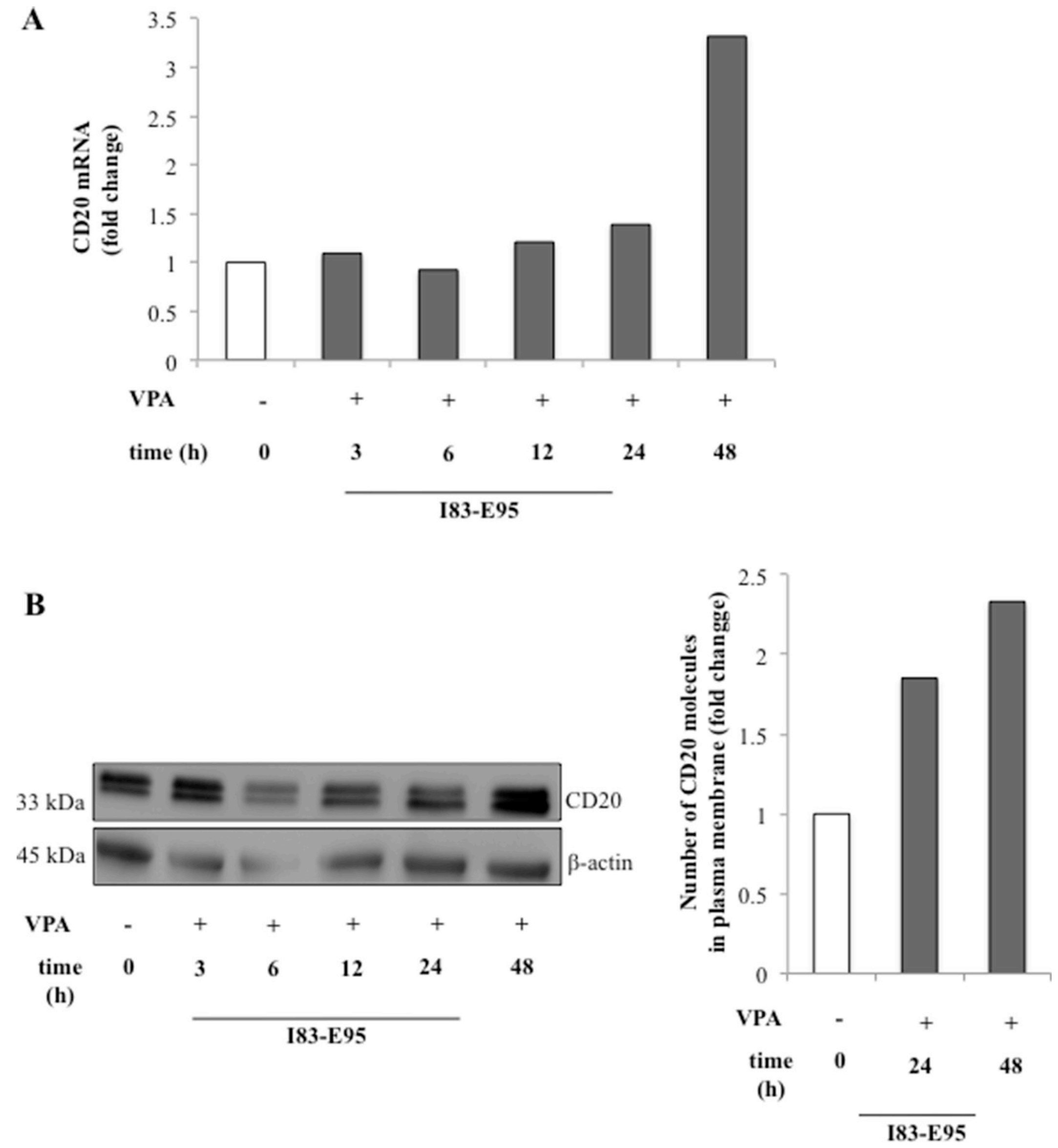

Supplementary Figure 2: CD20 is maximally upregulated in I83-E95 cells after 48 hours in response to valproate. I83-E95 cells were incubated with 1000 µM of valproate and mRNA and total protein lysates were collected at the indicated time points and further analysed by qPCR (**A**) and Western blot (**B**, left). Fold induction of CD20 on the plasma membrane as measured by Quantibrite beads after 24 and 48 h of incubation with valproate was analysed by FACS (**B**, right).
